# Effects of combined technology of micro-/nanobubble oxygenation irrigation and microbial agents on saline soil remediation and cotton yield

**DOI:** 10.3389/fpls.2025.1605834

**Published:** 2025-10-24

**Authors:** Qinyong Bian, Yongxia Yang, Liang Ma, Yingjie Ma, Zhiguo Wang, Yanbo Fu, Yaozu Feng, Jingquan Zhu, Yanhong Wei, Ling Lin

**Affiliations:** ^1^ College of Water Conservancy and Civil Engineering, Xinjiang Agricultural University, Urumqi, China; ^2^ Institute of Agricultural Resources and Environment, Xinjiang Academy of Agricultural Sciences, Urumqi, China; ^3^ College of Resources and Environment, Xinjiang Agricultural University, Urumqi, China; ^4^ Institute of Microbiology, Xinjiang Academy of Agricultural Sciences, Urumqi, China

**Keywords:** micro-/nanobubble oxygenation irrigation, microbial agents, cotton, saline soil, yield

## Abstract

Soil salinization severely limits Xinjiang cotton production sustainability, urgently requiring efficient, eco-friendly remediation. This study proposes a synergistic strategy of micro-nano bubble aeration + microbial agents (MA-MNBs) to improve saline-alkali soil via “physical aeration + biological regulation”. Field micro-plot experiments (completely randomized block, n=3) on Xinjiang saline-alkali cotton fields set 4 treatments: CK (conventional irrigation), MA (microbial agents), MNBs (micro-nano bubble irrigation), MA-MNBs (combination). Results showed: MA-MNBs significantly reduced soil salt by 26.08% vs CK (P<0.05); strongly activated soil enzyme activity (MA-MNBs > MA > MNBs); increased bacterial Chao1 index, enriched Proteobacteria/Actinobacteriota, and boosted Alternaria/Fusarium abundance; promoted cotton root growth, leaf area index, and dry matter accumulation (P<0.05); seed cotton yield reached 6278.65 kg·hm^-^² (44.53% higher than CK). Conclusion: MA-MNBs synergistically improves saline-alkali soil productivity by optimizing soil physicochemical and biological functions, providing an innovative solution for arid-region saline soil remediation and agricultural efficiency, as well as a theoretical basis for its mechanism and future application.

## 1 Introduction

Soil salinization has become one of the key issues constraining global agricultural production and ecological health (Kumar et al., 2024). As the largest cotton production base in China, more than one-third of the cultivated land in Xinjiang suffers from varying degrees of salinization, which poses serious constraints to local agricultural production (Zhang et al., 2025; Bai et al., 2024). Currently, saline-alkaline land management mainly relies on three approaches: physical improvement (Li D. et al., 2024);, chemical improvement (Elmeknassi et al., 2024), and biological improvement (Zheng et al., 2023), however, each of these methods has its own limitations, for example, physical improvement needs to consume a large amount of water resources, and chemical improvement may cause secondary pollution, whereas biological improvement is subject to environmental conditions. Therefore, there is an urgent need for an environmentally friendly, efficient and effective method to rehabilitate saline soils.

In recent years, the application of microbial agents (MA) in soil remediation has attracted much attention. Microbial agents show great potential in saline-alkaline land improvement by promoting plant nutrient uptake, enhancing metabolic functions and improving the ecological environment, while avoiding environmental pollution (Wu et al., 2024; Xing et al., 2024).The application of microbial agents enhances soil element transformation activity, accelerates litter decomposition, and facilitates the absorption or sequestration of salt ions. These agents secrete organic acids and/or macromolecule-degrading enzymes while providing organic matter to mitigate soil salinity and alkalinity. Furthermore, microbial inoculants improve the rhizosphere microenvironment, creating low-salinity/-alkalinity microzones conducive to plant growth, and produce phytohormones that stimulate plant growth or activate stress resistance systems. Additionally, they reduce the pH and EC of saline-alkali soils, increase microbial abundance, decrease soil salt concentrations, and elevate soil organic matter content, thereby promoting plant growth (Zhao et al., 2024; Dong et al., 2024). Micro-/nanobubble oxygenation irrigation are bubbles between micrometer and nanobubbles, with particle sizes ranging from 200 nm to 50 μm. Due to their small particle size, large specific surface area, and high pressure, MNBs are characterized by long storage time, strong aerosolization, and adsorption (Bian et al., 2025, 2024).MNBs enhance growth by improving soil aeration and optimizing oxygen availability, thereby promoting root respiration, mitigating oxidative damage to cell membranes induced by salt stress, and ameliorating plant physiological metabolism (Wang et al., 2021). Furthermore, MNBs stimulate microbial activity within the rhizospheric soil (Bian et al., 2024). The resultant active microbial community effectively decomposes soil humus and organic matter, accelerating soil carbon and nitrogen cycling. Concurrently, these microbes interact with soil minerals to form stable aggregates, thereby improving soil structure (Zhao and Hu, 2025). This structural enhancement increases soil permeability and aeration, further facilitating the leaching and migration of soluble salts and ultimately reducing salt stress (Zhang et al., 2024; Wang et al., 2025). While most existing research focuses on the individual effects of either micro-nano bubbles (MNBs) or microbial agents (MA) for ameliorating saline-alkali soils, a limited body of work posits that their synergistic interaction enhances crop rhizosphere conditions, thereby creating a more favorable habitat for soil microorganisms. Crucially, microbial agents exhibit heightened activity within oxygen-enriched environments generated by MNBs. This synergy ultimately fosters a more conducive micro-ecosystem for microbial community proliferation and root growth, contributing to concurrent improvements in crop yield and quality (Zhang et al., 2024). Nevertheless, the precise mechanisms underlying how the MA-MNBs synergy ameliorates saline-alkali soils and promotes crop yield enhancement remain poorly understood.

In this study, the cotton field in Xinjiang was used as the research object to investigate the effects of the synergistic effect of MA and MNBs on soil salinity, enzyme activity, microbial community structure, as well as cotton growth and yield in the cotton field. By comparing the response characteristics of cotton field soil environment and crops under different treatments, we aimed to reveal the synergistic mechanism of MA and MNBs on saline soil improvement, and provide new ideas and technical support for saline land management and agricultural production.

## 2 Materials and methods

### 2.1 Experimental site

**Figure 1:**
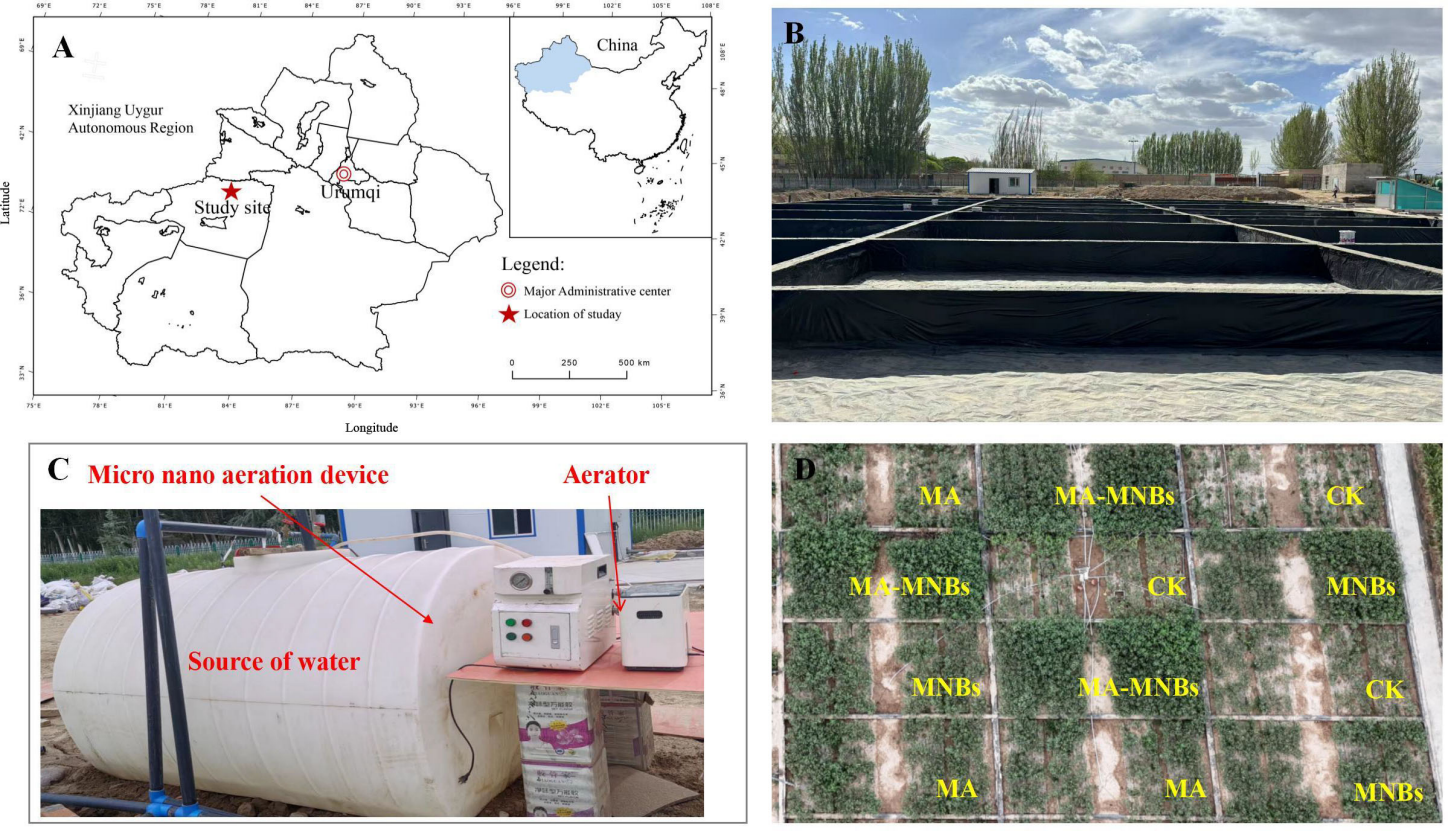
Experimental location and schematic diagram of the experimental setup. **(A)** location and layout of the study area; **(B)** photographs of salt ponds; **(C)** irrigation equipment; **(D)** schematic diagram of the experimental design.

The experiment was conducted from April to November 2024 at the Aksu Field Scientific Observatory and Research Station for Soil Quality of the Ministry of Agriculture and Rural Affairs (41°48′N, 81°54′55″E) at an altitude of 1200 m (**Figure 1A**). The climate was temperate continental arid, with an average annual temperature of 7.6°C, a maximum temperature of 38.3°C, a minimum temperature of -28°C, a frost-free period of 133-163 d, an average annual sunshine hours of 2789.7 h, and an average annual precipitation of 171.13 mm. The soil was clayey, with a pH value of 7.91, a bulk density of 1.43 g**·**cm^-3^, water-soluble salinity of 0.07 g**·**kg^-1^, and hydrolyzed soluble salts 0.7g**·**kg^-1^, hydrolyzed nitrogen 81.4 mg**·**kg^-1^, effective phosphorus 14.7 mg**·**kg^-1^, quick-acting potassium 171 mg**·**kg^-1^, organic matter 13.66mg**·**kg^-1^.

### 2.2 Experimental design

The experimental soils were primarily collected from two sources: the surface layer (0–20 cm depth) of agricultural soil at the Aksu Field Scientific Observation and Research Station for Soil Quality, Ministry of Agriculture and Rural Affairs (EC=0.12 dS/m, salt content=0.7 g/kg), and the topsoil layer (0–5 cm depth) of natural saline soil formed on the Gobi desert in Alaer City (EC=69 dS/m, salt content=480 g/kg). To achieve the target salinity level (moderately saline-alkali soil) specified in the experimental design, the two soil types were blended in specific proportions and repeatedly mixed to produce a homogenized soil mixture (EC=5.93 dS/m, salt content=6.4 g/kg). This mixture was then packed into designated micro-plots within the salt pool. According to the Chinese National Standard General Techniques for Saline-Alkali Land Improvement (GB/T 42828.3-2023), this prepared soil is classified as moderately saline-alkali soil. The test cotton variety was “J026-5”(provided by Xinjiang Academy of Agricultural Sciences), which was manually sown on May 7, 2024, and the sampling was completed on October 15th. The planting pattern was “1 film, 3 tubes and 6 rows”, with wide rows of 66 cm, narrow rows of 10 cm, and plant spacing of 11 cm.

The experiment was conducted in micro-zones of salt ponds, and the size of a single salt pond was 10 m (length) × 5 m (width) × 1 m (height) (**Figure 1B**). A completely randomized block design was used with four treatments: conventional water irrigation (CK), microbial agents (MA), micro-/nanobubble oxygenation irrigation (MNBs), and micro-/nanobubble oxygenation irrigation + microbial agents group (MA-MNBs), each with three replications (**Figure 1C**). Prior to the experiment, salt content measurements were conducted on the three replicate plots, with results showing that the coefficient of variation was consistently below 5%, indicating weak variability. Conventional water was utilized from surface water in the project area with a dissolved oxygen (DO) concentration of 8-9 mg/L. micro-/nanobubble oxygenation irrigation water was prepared using a B&W micro-nano bubble generator (produced by Benzhou (Beijing) New Technology Promotion Co.,Ltd., Beijing, China with a working pressure of 0.015 MPa and an inlet flow rate of 1.5 L/min). In order to enhance the DO content in the irrigation water, an oxygen supply device (Jiangsu Yuyue Medical Equipment Co., Ltd., DanYang, China, YU300 type,oxygen flow rate of 2 L/min) was utilized, set to the maximum amount of oxygen supply (oxygen supply concentration of 90%), and connected with the B&W micro-nano bubble generating device. The B&W micro-nano bubble generating device was operated to stabilize the changes in dissolved oxygen, and then the HQ40 type was employed. A portable dissolved oxygen meter (Seven 2 Go™, METTLER TOLEDO INTERNATIONAL TRADING CO., LTD., Shanghai, China,±0.1 mg/L) was employed to monitor the dissolved oxygen concentration content of the water body (DO=30 mg/L). The micro-/nanobubble oxygenation irrigation water was transported to the root zone of the crop through the drip irrigation system(**Figure 1D**). The liquid microbial agents (containing Bacillus subtilis strain SJA-15 and Trichoderma harzianum strain SJA-32 at concentrations of 1×10^^8^ CFU/mL and 5×10^^7^ CFU/mL respectively) were produced by Shujian Agricultural Environmental Protection Technology Co., Ltd. (Urumqi, China). The irrigation quota and fertilization program during the whole life cycle were implemented with reference to the local conventional cultivation management standards.

### 2.3 Measurement and application methods

#### 2.3.1 Cotton growth and yield

Root length: Three cotton plants were randomly selected from each treatment at maturity and profiled by deep excavation at 10 cm from the main stem and 60 cm vertically, after repairing neatly, the retrieved root samples were rinsed and wiped dry, and then imaged using a scanner (EPSON Expression 10000XL), and root phenotypic information (root length) was obtained using root system analysis software (WinRhizo REG 2009, Canada) to obtain root phenotypic information (root length).

Leaf area index (LAI) and dry matter mass (DM): Three representative cotton plants were selected from each plot, and their leaves were separated and spread on a white plate, and then scanned with image software to obtain the LAI after taking pictures with a camera in jpg format. Cotton roots, stems, leaves and reproductive organs were separated, then killed at 105 °C for 30 min, dried at 75 °C until constant weight, and weighed for dry matter accumulation after cooling (Escobar Diaz et al., 2021).

Yield: The yield of cotton was determined at the end of maturity by the whole-harvest method, recording the total number of bolls with a diameter of more than 2 cm in each treatment, and picking 100 bolls in each sample plot and weighing them to calculate the mass of a single boll. Seed cotton yield is the product of total number of bolls and boll mass (Jin et al., 2024).

#### 2.3.2 Soil salinity

Adopting 5-point sampling method, respectively, in the cotton seedling stage, bud stage, flowering stage, maturity stage in the mulch with soil auger randomly take soil samples from 0 to 20 cm soil layer, mixed homogeneous samples to bring back to the laboratory, soil samples after drying and grinding through a 2 mm sieve weighed 20 g, the soil and deionized water according to the quality of the ratio of 1:5 mixing, with the shaker or shaking table shaking for 0.5 h, the use of the mass method of determining the soluble salt content of the soil (Kargas et al., 2018).

#### 2.3.3 Soil enzyme activity and microbial sequencing

Several studies have found that microbial diversity increases and microbial community distribution becomes more homogeneous during crop maturation (Hu et al., 2020). Therefore, inter-root soil samples were collected during cotton maturation and three cotton plants were randomly selected for sampling in each treatment. Rectangular soil blocks (20 cm × 20 cm × 40 cm) were cut vertically along the cotton root system. Loose soil was then removed by gentle shaking, and soil adhering to the root surface was brushed off and placed in sterile bags and promptly transported back to the laboratory. The soil was divided into two parts for subsequent analysis. The first portion was placed in a refrigerator for determination of soil enzyme activity. The second portion was stored in the refrigerator at -80 °C for subsequent sequencing of soil microorganisms. The rhizosphere soil samples were analyzed by the Soil Testing Laboratory of Xinjiang Academy of Agricultural Sciences.

##### 2.3.3.1 Measurement of soil enzyme activity

Using the Tabatabai method (Urlić et al., 2023), soil alkaline phosphatase activity (ALP) was estimated by determining the activity of nitrophenyl phosphate (PNPP) released at 410 nm. Soil sucrase (SU) was determined by the 3,5-dinitrosalicylic acid (DNS) method to determine the reducing sugar content and absorbance was measured at 540 nm after color development. Soil catalase was used to decompose H_2_O_2_ to produce H_2_O and O_2_, and the enzyme activity was calculated by titrating the amount of residual H_2_O_2_ with potassium permanganate; urease (URE) was used to develop color by Nye’s reagent with NH3, and the absorbance was measured at 630 nm (Alef and Nannipieri, 1995).

##### 2.3.3.2 PCR amplification and highthroughput sequencing

Fresh rhizosphere soil samples (0.5 g) were weighed, and microbial DNA was extracted using the E.Z.N.A.^®^ Soil DNA Kit (Omega Bio-tek, Norcross, GA, U.S.). DNA quality and quantity were determined using a NanoDrop 2000 Spectrophotometer (Bio-Rad Laboratories Inc., USA).

For bacterial community analysis, primers 338F (5’-ACTCCTACGGGAGGCAGCA-3’) and 806R (5’-GGACTACHVGGGTWTCTAAT-3’) targeting the V3-V4 region of 16S rRNA were used, while fungal ITS1 regions were amplified with primers ITS5F (5’-GGAAGTAAAAGTCGTAACAAGG-3’) and ITS1R (5’-GCTGCGTTCTTCATCGATGC-3’). The 25 µL PCR system contained:5 µL 5×reactionbuffer, 4 µL 5× GC buffer, 2 µL 2.5 mM dNTPs, 1 µL each forward/reverse primer (10 mM), 2 µL DNA template, 0.25 µL Q5 Hi-Fi DNA polymerase, and ddH_2_O to adjust the total volume. Ampl-ification conditions followed: 95 °C predenaturation (2 min); 25-30 cycles of 98 °C denaturation (15s), 55 °C annealing (30 s), and 72 °C extension (30 s); with final extension at 72 °C (5 min).

Amplification products were sequenced using the Illumina MiSeq PE300 platform. Raw sequencing data underwent quality control with fastp (Chen et al., 2018) (version 0.20.0; https://github.com/OpenGene/fastp). Overlapping reads were assembled into tags using FLASH (Magoč and Salzberg, 2011) (version 1.2.7; https://www.flash.cn). Trimmomatic software (version 0.33) filtered low-quality tags to obtain high-quality sequences. UPARSE (Edgar, 2013) (version 7.1; http://drive5.com/uparse/) clustered non-repetitive sequences into operational taxonomic units (OTUs) at 97% similarity (Stackebrandt and Goebel, 1994), with removal of chimeras and singletons during clustering. Optimized sequences were mapped to OTU representative sequences using ≥97% similarity thresholds. Microbial diversity and community composition analyses were performed separately for bacterial and fungal datasets.

### 2.4 Statistical analysis

All data were preprocessed using Excel 2016 (Microsoft, Redmond, USA), SPSS Statistics 27 (IBM, Armonk, USA), and Origin 2021 (OriginLab, Northampton, USA).One-way ANOVA was employed to assess the significant differences in soil salinity, enzyme activity, and microbial community diversity indices among different treatment groups. A significance level of α=0.05 was set, and F-tests were conducted to determine the intergroup differences. The experimental area was mapped using ArcGIS software. Bar graphs were utilized to show the effect of different treatments on soil salinity. Box plots were utilized to show changes in soil enzyme activities and soil microbial community diversity indices for different treatments, and stacked bar plots were utilized to show microbial community composition.

## 3 Results

### 3.1 Effects of different treatments on soil salinity

**Figure 2:**
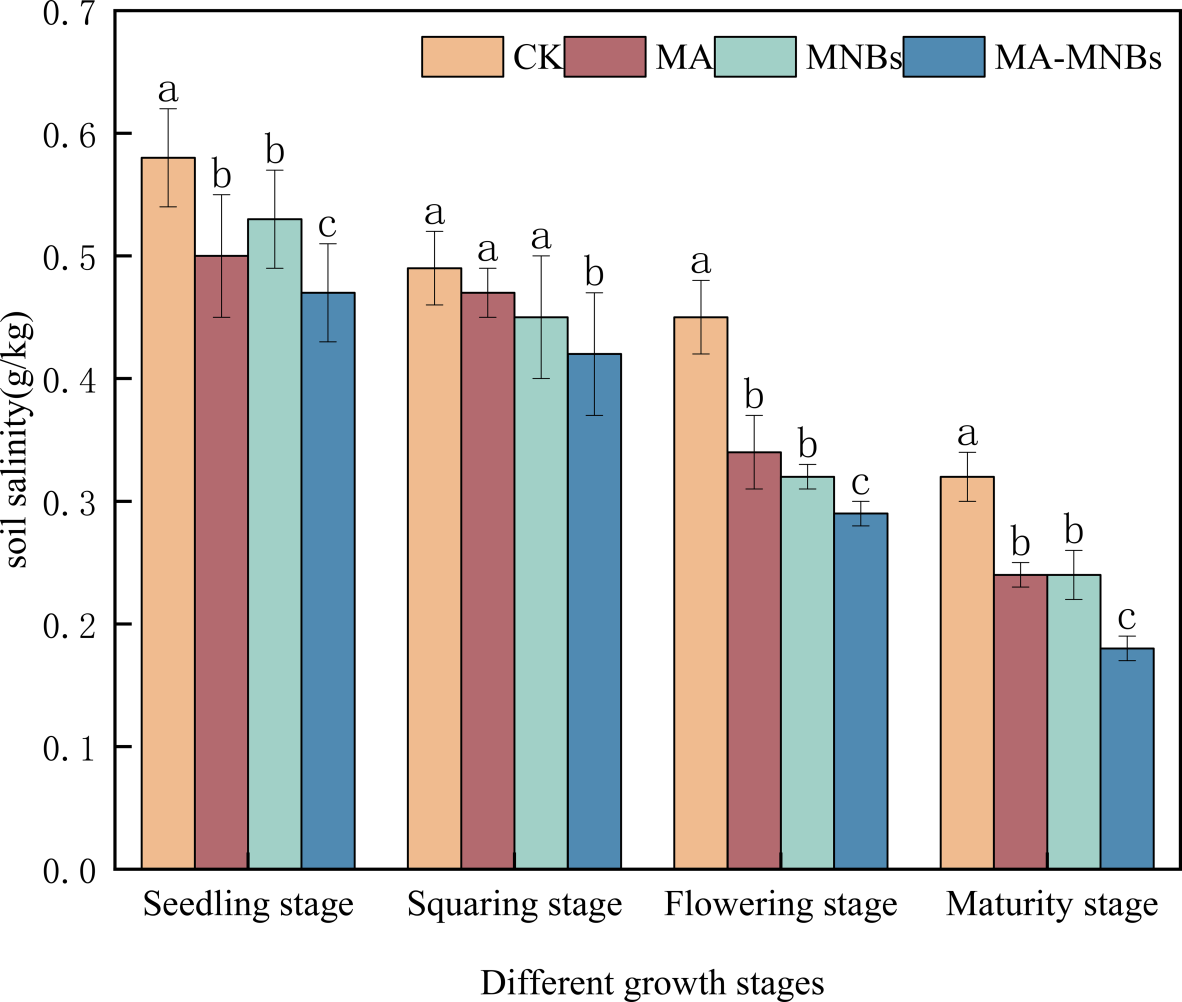
Effects of different treatments on soil salinity in cotton. Different lower case letters (a, b, c,…) indicate significant differences (*P*<0.05) in the mean soil salinity of different treatments at different fertility stages of cotton, and the same lowercase letters indicate that there were no significant differences (*P* > 0.05) in the mean soil salinity of different treatments at different fertility stages of cotton; vertical bars indicate the mean±standard deviation (SD) based on three replications.

Soil salinity of the four different treatments gradually decreased over time (**Figure 2**). Compared with CK, the soil salinity of the three different treatments (MA, MNBs and MA-MNBs) decreased by 15.76%, 16.36% and 26.08%(*P*<0.05), respectively. indicating that the single use of microbial agents or micro-/nanobubble oxygenation irrigation has the effect of improving soil salinity, especially the combined effect of microbial agents or micro-nano-bubble oxygenation irrigation is more obvious.

### 3.2 Effects of different treatments on soil enzyme activities

**Figure 3:**
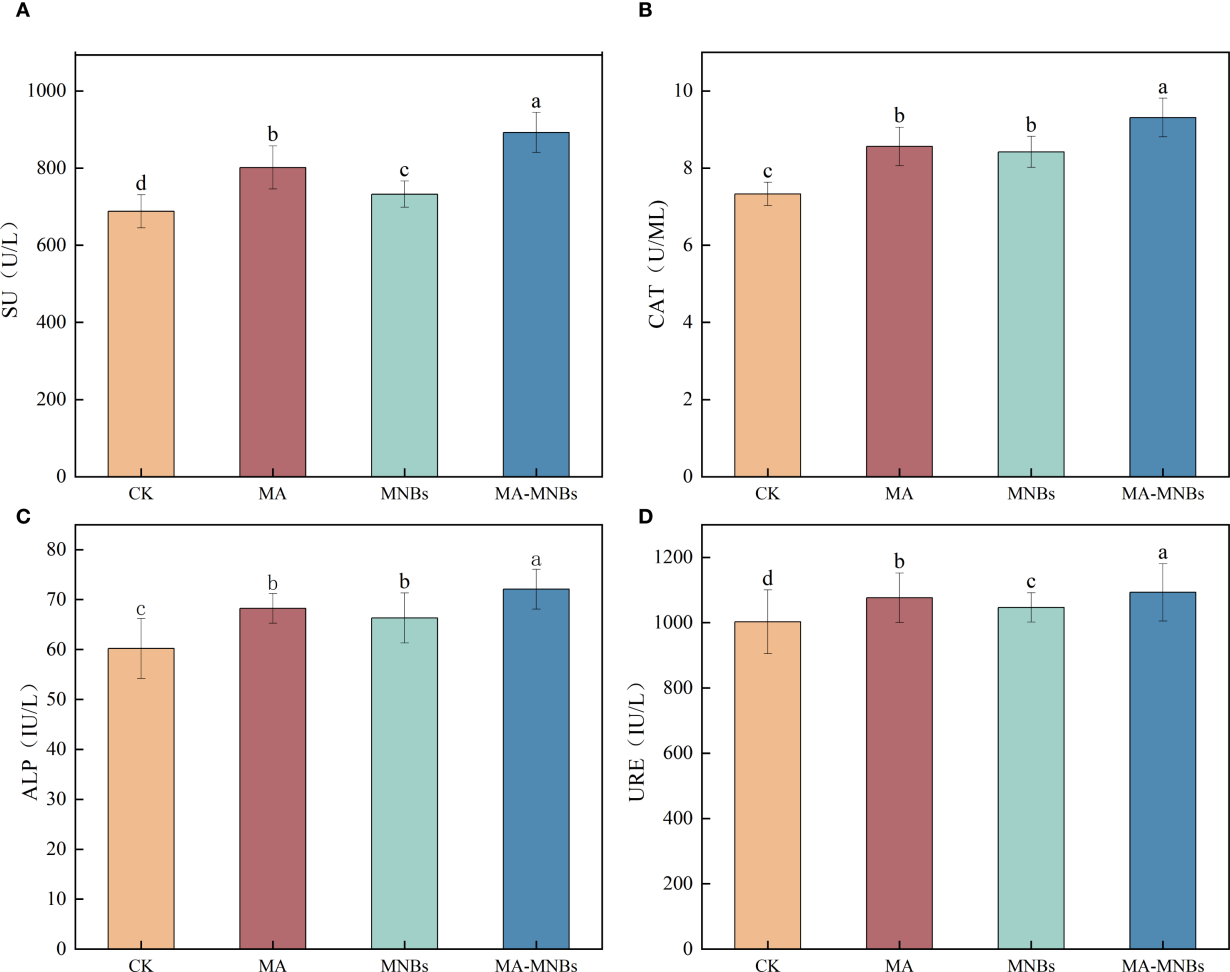
Effects of different treatments on soil enzyme activities in cotton. **(A)** soil sucrase; **(B)** catalase; **(C)** alkaline phosphatase; **(D)** urease.Different lowercase letters (a, b, c,…) indicate significant differences (*P*<0.05) in the mean values of soil enzyme activity contents under different treatments, and the same lowercase letters indicate no significant differences (*P* > 0.05) in the mean values of soil enzyme activity contents under different treatments; vertical bars indicate the mean±standard deviation (SD) based on three replications.

To investigate the effects of different treatments on soil enzyme activities in cotton (**Figure 3**), this study analyzed the response characteristics of soil sucrase (SU), catalase (CAT), alkaline phosphatase (ALP) and urease (URE) activities under four treatments. The data demonstrate that MA-MNBs, MA, and MNBs treatments significantly increased all four soil enzyme activities compared to CK treatment (*P*<0.05), with synergistic effects following the order: MA-MNBs > MA > MNBs > CK. Among them, compared with CK, soil sucrase, soil catalase, soil alkaline phosphatase, and soil urease activities were increased by 29.76%, 27.01%, 19.76%, and 9.04%, respectively, in MA-MNBs treatment (*P*<0.05); and 16.56%, 16.56%, 16.78%, 13.35%, and 13.35%, respectively, in MA treatment, 16.78%, 13.35%, and 7.37% (*P*<0.05), respectively; and soil sucrase, soil catalase, soil alkaline phosphatase, and soil urease activities were increased by 6.48%, 14.87%, 10.13%, and 4.42% (*P*<0.05), respectively, in MNBs treatment. It indicated that the synergistic effect of the two amendments had a specific enhancement effect on the activation of carbon and nitrogen cycle related enzyme systems.

### 3.3 Effects of different treatments on soil microbial community structure

#### 3.3.1 Microbial community diversity

**Figure 4:**
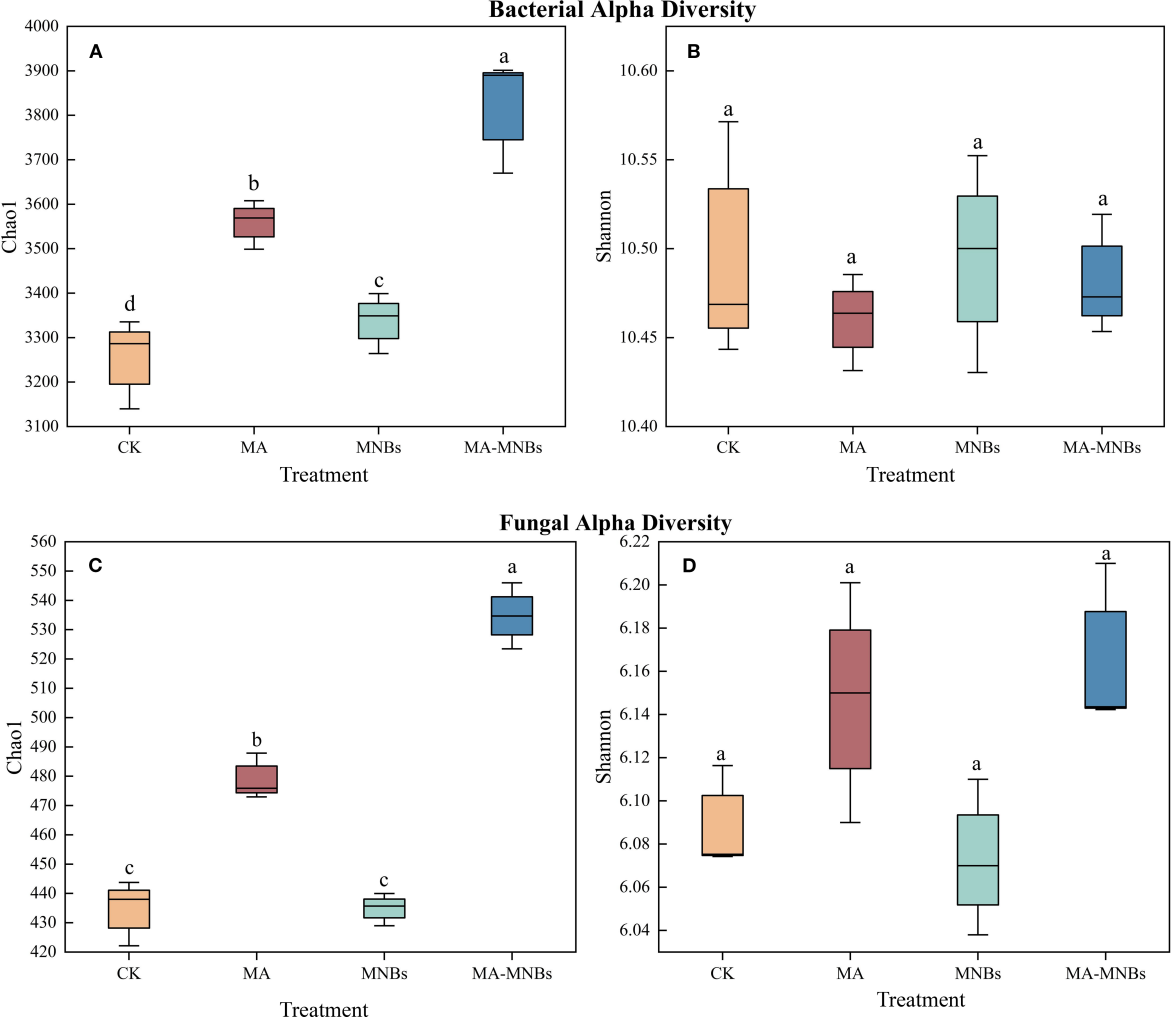
Effects of different treatments on soil microbial community diversity.Bacterial alpha diversity is represented by the Chao1 index **(A)** and the Shannon index **(B)**. Similarly, fungal alpha diversity is represented by the Chao1 index **(C)** and the Shannon index **(D)**.The boxplots display the data for each treatment, with the boxes representing the interquartile range (IQR), the horizontal line inside the box indicating the median, and the whiskers extending to 1.5 times the IQR.Different lower case letters (a, b, c,…) indicate significant differences (p<0.05) in the mean values of soil enzyme activity contents under different treatments, and the same lowercase letters indicate that there are no significant differences (*P* > 0.05) in the mean values of soil enzyme activity contents under different treatments; vertical bars indicate the mean±standard deviation (SD) based on three replications.

In this study, the effects of four different treatments on bacterial and fungal diversity were analyzed (**Figure 4**). It can be seen that MA-MNBs, MA and MNBs treatments significantly enhanced the bacterial Chao 1 index compared to CK treatment(*P*<0.05), but there was no significant difference in the bacterial Shannon index among the different treatments (*P*>0.05). Similarly, MA-MNBs, MA treatments significantly fungal Chao 1 index(*P*<0.05), but there was no significant difference between CK and MNBs treatments (*P*>0.05); there was no significant difference in fungal Shannon index between treatments.

#### 3.3.2 Microbial community structure

**Figure 5:**
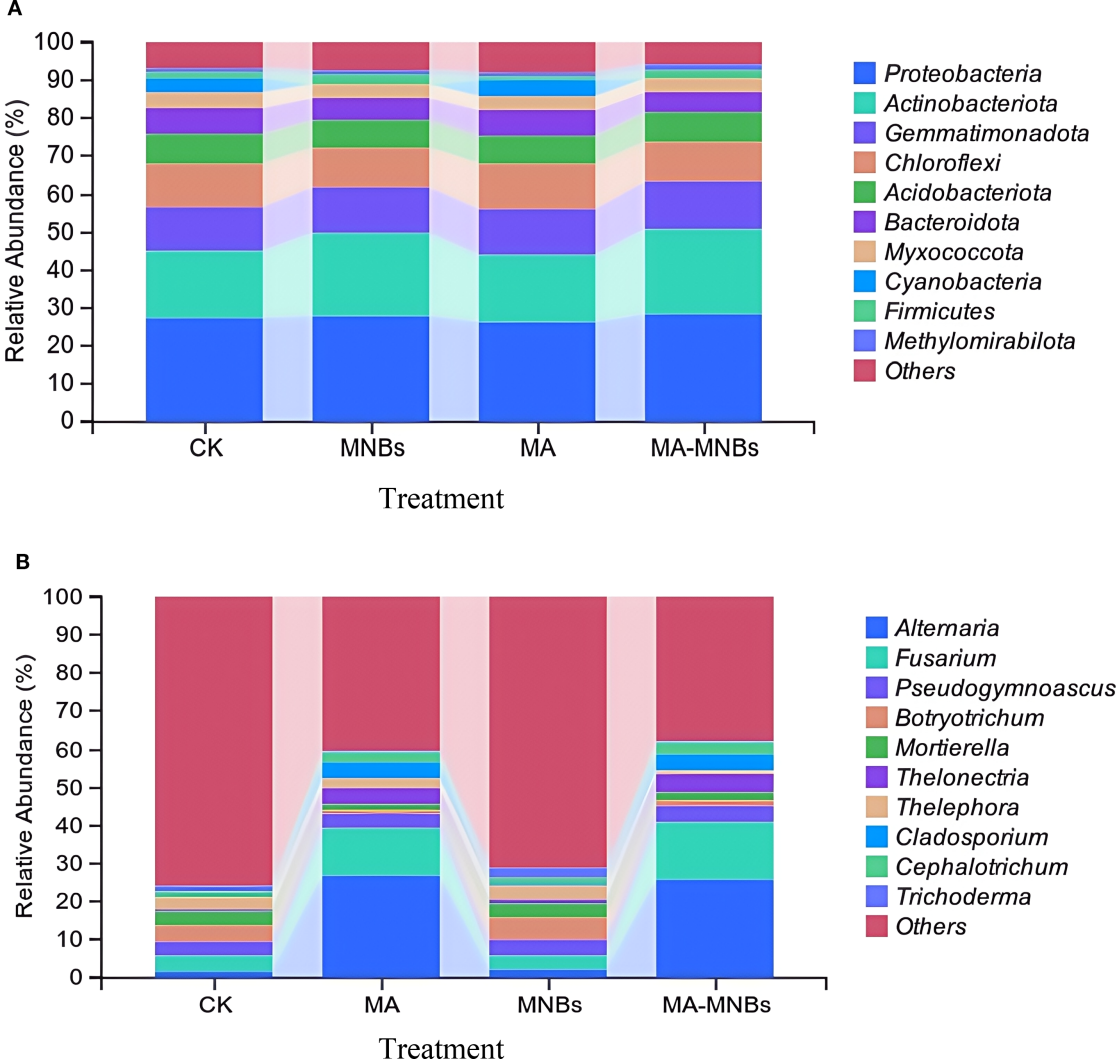
Effects of different treatments on soil microbial community structure. **(A)** soil bacterial community structure at genus level (TOP10); **(B)** soil fungal community structure at genus level (TOP10).

The top10 species in abundance were selected from the soil samples of different treatments for presentation and the other species were categorized as Others (**Figure 5**). The relative abundance of dominant genera of bacteria with more than 5% in different treatments was in the order of size of Proteobacteria, Actinobacteriota, Gemmatimonadota, Chloroflexi, Acidobacteriota, and 6 species of Bacteroidota, but overall the dominant genera among the different treatments Relative abundance differences were small (**Figure 5A**), but MA-MNBs and MNBs treatments increased Proteobacteria, Actinobacteriota to a certain extent. the relative abundance of dominant genera of fungi with more than 5% in different treatments was Altemaria, Fusarium in order of magnitude; at the genera level between CK and MNBs treatments Microbial abundance differences were small, but the two treatments MA-MNBs and MA significantly increased Altemaria and Fusarium abundance, and the effect of MA-MNBs was more obvious(**Figure 5B**).

### 3.4 Effects of different treatments on cotton growth

**Figure 6:**
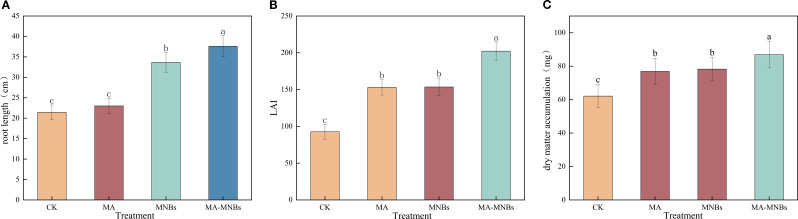
Effects of different treatments on cotton growth. **(A)** root length; **(B)** leaf area index; **(C)** dry matter accumulation. Different lowercase letters (a, b, c,…) indicate significant differences (*P*<0.05) in the mean values of cotton growth indexes under different treatments, and the same lowercase letters indicate no significant differences (*P* > 0.05) in the mean values of growth indexes under different treatments; vertical bars indicate the mean±standard deviation (SD) based on three replications.

The effects of four treatments on cotton root length, leaf area index (LAI), and dry matter accumulation were analyzed in this study (**Figure 6**). The results demonstrate that MA-MNBs and MNBs treatments significantly increased cotton root length by 75.45% and 56.99%, respectively, compared to the CK treatment (p<0.05). In contrast, the MA treatment showed no significant effect (*P*>0.05). This differential response may be attributed to the improved root zone oxygen environment through oxygenated irrigation, which enhances root metabolic activity. Furthermore, the addition of microbial fungicides appears to have a more pronounced effect on root system improvement. For leaf area index (LAI), the three different treatments (MA-MNBs, MA and MNBs) increased 118.35%, 65.14%, and 65.78%, respectively, compared with the CK treatment, with no significant difference (*P*>0.05) for MA and MNBs. For dry matter accumulation, three different treatments (MA-MNBs, MA and MNBs) increased 39.82%, 23.76%, and 26.00%, respectively, compared to CK treatment, where MA and MNBs did not differ significantly (*P*>0.05). Micro-/nanobubble oxygenation irrigation improved the oxygen environment in the cotton root zone and enhanced root respiration, while microbial agents secreted metabolites that facilitated photosynthetic product accumulation. These synergistic interactions established a “oxygen–bacteria mutual promotion” cycle, thereby exerting more pronounced effects on cotton root elongation, leaf area index, and dry matter accumulation.

### 3.5 Effect of different treatments on cotton yield

The effect of four treatments on yield components was analyzed in this study (**Table 1**). The three experimental treatments (MA, MNBs, and MA-MNBs) demonstrated yield increases of 10.10%, 20.08%, and 44.53%, respectively, relative to the CK treatment(*P*<0.05). These results suggest that aerobic irrigation alone had a greater impact on yield enhancement than microbial fungicide application alone, while the combination of both factors (MA-MNBs) produced a significant synergistic effect.

**Table 1:** Effects of different treatments on cotton yield.

Treatments	Number of bolls per plant	Weight per boll/g	Seed cotton yield/ (kg·hm^-2^)	lint yield (kg·hm^-2^)
CK	8.01±0.67d	4.14±0.16d	4344.26±178.59d	1086.06±70.88d
MA	8.26±0.73c	4.89±0.18c	4782.92±251.11c	1195.73±82.19c
MNBs	9.34±0.98b	5.06±0.2b	5216.49±297.19b	1304.12±69.76b
MA-MNBs	9.98±1.34a	6.52±0.26a	6278.65±326.14a	1569.66±78.34a

Data are expressed as mean±standard deviation (SD) of three replications. Different lower case letters (a, b, c,…) indicate significant differences (*P*<0.05) in the mean cotton yield under different treatments, and the same lower case letters indicate no significant differences (*P* > 0.05) in the mean cotton yield under different treatments.

### 3.6 Correlation analysis

**Figure 7:**
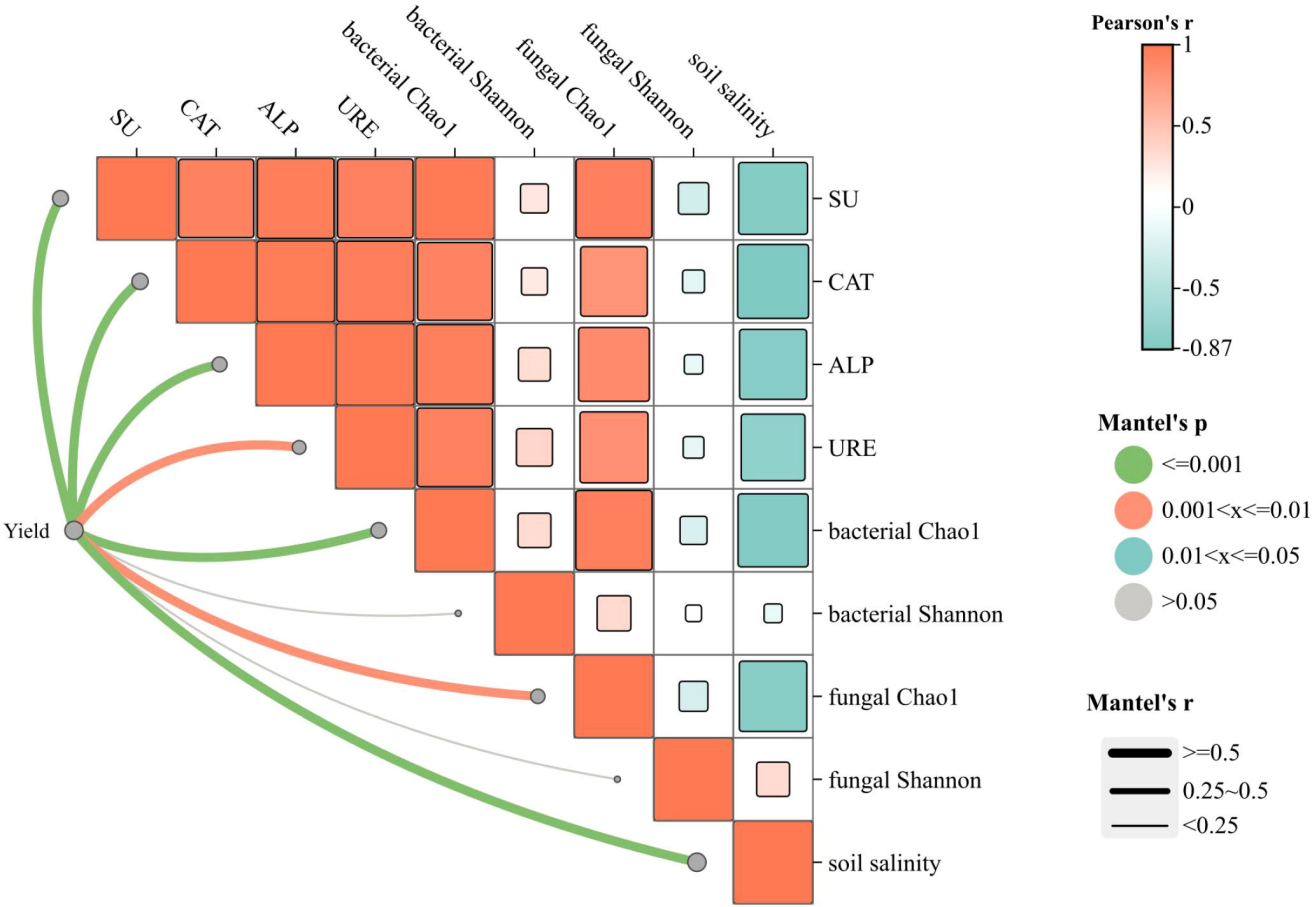
Correlation analysis between yield and other indicators. Orange bands represent positive correlations and light blue negative correlations. The width of the line indicates the magnitude of the absolute value of the correlation (Mantel’s r) and the color of the line indicates the range of the significance p-value (Mantel’s p).

The Mantel test (based on the Bray-Curtis distance matrix) was employed to analyze the correlations between cotton yield and other indicators (**Figure 7**). The results revealed that soil salinity exhibited significantly negative correlations with the activities of four enzymes, bacterial Chao1 index, and fungal Chao1 index (Pearson’s r=-0.5 to -0.97, P<0.05), whereas no statistical significance was observed between soil salinity and bacterial or fungal Shannon index (Pearson’s r=0 to 0.5, P > 0.05). Additionally, cotton yield showed significantly positive correlations with soil salinity, activities of the four enzymes, bacterial Chao1 index, and fungal Chao1 index (Pearson’s r=0.5 to 1, P<0.01), while no significant correlation was found between yield and Shannon index (P > 0.05).

## 4 Discussion

This study revealed that the combined application of microbial agents (MA) and micro-/nanobubble oxygenation irrigation (MNBs) exhibits a synergistic mechanism for saline soil improvement. Among all treatments, MA-MNBs co-treatment achieved the most significant reduction in soil salinity, outperforming individual MA or MNBs applications. While MA alone reduced salinity via organic acid production (e.g., oxalic acid, citric acid) that chemically neutralizes soil salts (Xue et al., 2024; Mao et al., 2022), MNBs independently enhanced salt leaching by improving soil pore structure and water infiltration efficiency (Du et al., 2023). The synergistic effects between (MNBs and MAin saline soil remediation arise through integrated chemical, physical and biological mechanisms: chemically, organic acids (e.g., oxalic and citric acids) secreted by MA not only neutralize salt ions but also disrupt soil salt bridges via their carboxyl groups, modifying local electrochemical gradients, while oxygen radicals generated by MNBs oxidize salt crystal surfaces to enhance ion dissociation efficiency; physically, MNBs improve soil pore connectivity through cavitation effects and MA hyphal networks stabilize pore architecture, collectively enhancing salt leaching; biologically, MNBs optimize rhizosphere oxygen conditions to stimulate proliferation and metabolic activity of aerobic microorganisms while promoting root exudation of organic carbon sources (e.g., malate), with MA functional strains synergistically activating electron transport chains to boost microbial energy metabolism, ultimately forming protective biofilms that reduce rhizosphere Na^+^ concentration and improve crop root development and biomass accumulation. The synergistic effect likely arises from MA metabolites accelerating salt ion migration, while MNBs amplify ion exchange and leaching by oxygenating the rhizosphere, aligning with previous reports on microbial-physical synergy in saline soil remediation (Zhang et al., 2024).

Soil enzyme activities were more sensitive to different treatments, and the activation effects of MA-MNBs treatments on key enzymes of the cycle, such as sucrase (SU) and catalase (CAT), were significantly better than those of single treatments, and the strength of the synergistic effect showed a gradient pattern of MA-MNBs > MA > MNBs. This phenomenon may be attributed to the direct introduction of functional flora by microbicides to stimulate enzyme synthesis (Caldara et al., 2024), whereas aerobic irrigation mitigates the inhibition of enzyme activities by anaerobic environment by improving the inter-root oxygen content (Li Y. et al., 2024). Therefore, the synergistic effect of microbial agents and micro- and nano-bubble oxygenated irrigation can specifically enhance the activities of soil-related enzyme systems and optimize soil nutrient transformation and supply processes. Microbial diversity analysis showed that the MA-MNBs treatment significantly increased the bacterial Chao1 index but did not change the Shannon index, indicating that the MA - MNBs co-treatment optimized the microbial community structure mainly by increasing species richness. Specifically, the enrichment of the dominant bacterial phylum Proteobacteria, Actinobacteriota in the MA-MNBs treatment may be related to the improved oxygen environment of micro- and nano-bubbles, which have stronger metabolism under aerobic conditions (Ouyang et al., 2021). The present study showed that MNBs treatment alone had no significant effect on fungal Chao1 index, suggesting that the regulation of fungal communities by microbubble oxygenation is dependent on the synergistic effect of mycorrhizal fertilizers. At the genus level, although the difference in Alternaria and Fusarium abundance between the control CK and MNBs treatments was not significant, microbial agents (MA) and their combined treatments with MNBs (MA-MNBs) significantly elevated the abundance of both. on the one hand, MA may, through the addition of functional mycorrhizae and exogenous organic matter (Liu et al., 2025; Yang et al., 2023), directly activating the metabolic activities of Alternaria and Fusarium; on the other hand, MNBs were further enhanced by optimizing the aerobic metabolic environments of the fungi on the basis of the addition of microbial agents, which both provided metabolic substrates for the fungi and improved the redox conditions (Xue et al., 2022; Zhang et al., 2023), further providing a better environment for Alternaria and Fusarium to create advantageous ecological niches. It is noteworthy that fungi of the genera Alternaria and Fusarium exhibit dual functions in agricultural ecosystems. Certain strains may promote plant root development by secreting growth hormones (e.g., IAA), while other species within the same genera can induce crop diseases and produce genotoxic toxins. The specific functions of the strains with increased abundance in this study require further confirmation through isolation and identification.

The efficacy of MA-MNBs treatment on cotton root length, leaf area index and dry matter accumulation was significantly better than that of single treatment, which confirmed the systematic regulation of plant physiological processes by “oxygen-bacteria mutual promotion”. Meanwhile, the cotton yield of MA-MNBs treatment was 44.53% higher than that of CK, which was significantly higher than that of single treatment. The results of Mantel test showed that soil salinity was significantly negatively correlated with the activities of four key enzymes (urease, sucrase, dehydrogenase, alkaline phosphatase), bacterial Chao 1 index and fungal Chao 1 index, indicating that salinity stress has a dual inhibitory effect on inter-root functions by suppressing soil microbial community abundance and enzyme activities. This indicates that salt stress has a dual inhibitory effect on inter-root functions by suppressing soil microbial community richness and enzyme activities (Li et al., 2021; Li D. et al., 2024). Salinity had no significant effect on microbial diversity (Shannon index), suggesting that salt stress mainly reduced microbial population size rather than altered community evenness, which is consistent with the results of some scholars (Chen et al., 2024; Zheng et al., 2024). Cotton yield was highly significantly and negatively correlated with soil salinity, while it was significantly and positively correlated with enzyme activity, bacterial Chao 1 index and fungal Chao 1 index. This suggests that synergistic restoration of microbial community abundance and functional activity is the core pathway for yield enhancement in saline soils: while the non-significant correlation between Shannon’s index and yield suggests that restoration of microbial species number is prioritized over community structural equilibrium in saline soil amelioration, a finding that provides theoretical justification for a targeted microbial inoculation strategy.

## 5 Conclusions

Based on micro-plot experiments, we confirmed that the combination of micro-/nanobubble oxygenation irrigation (MNBs) and microbial agents (MA) significantly improved the saline soil environment and cotton productivity, effectively reduced soil salinity, activated soil enzymes, optimized the structure of the inter-root microbial community, and thus promoted the development of the cotton root system and the accumulation of photosynthesis, which ultimately led to a significant increase in the cotton yield. The combination of MNBs and MA breaks through the bottleneck of the traditional saline soil improvement techniques, such as high water consumption, easy pollution or low efficiency. The combination of MNBs and MAs breaks through the bottlenecks of traditional saline soil improvement techniques, such as high water consumption, easy pollution or low efficiency, and provides a solution for saline soil restoration and high crop yield in arid areas. In the future, we can combine the multi-omics technology to analyze the molecular mechanism of the interaction between key functional bacteria and the oxygen environment, and further optimize the synergistic technology system.

## Data Availability

The data presented in the study are deposited in the SRA repository, accession number: SRP627859.
